# Systemic Epstein-Barr-virus-positive T cell lymphoproliferative childhood disease in a 22-year-old Caucasian man: A case report and review of the literature

**DOI:** 10.1186/1752-1947-5-218

**Published:** 2011-06-07

**Authors:** Valentina Tabanelli, Claudio Agostinelli, Elena Sabattini, Anna Gazzola, Francesco Bacci, Saveria Capria, Claudia Mannu, Simona Righi, Maria Teresa Sista, Giovanna Meloni, Stefano A Pileri, Pier Paolo Piccaluga

**Affiliations:** 1 Department of Hematology and Oncological Sciences 'L and A Seràgnoli', Hematopathology Section, S Orsola-Malpighi Hospital, University of Bologna, Bologna, Italy; 2 Hematology, Department of Cellular Biotechnologies and Hematology, 'Sapienza' University, Rome, Italy

## Abstract

**Introduction:**

Systemic Epstein-Barr-virus-positive T cell lymphoproliferative disease of childhood is an extremely rare disorder, characterized by clonal proliferation of Epstein-Barr-virus-infected T cells with an activated cytotoxic phenotype. The disease is more frequent in Asia and South America, with only few cases reported in Western countries. A prompt diagnosis, though often difficult, is a necessity due to the very aggressive clinical course of the disease.

**Case presentation:**

We report the clinicopathological features of fulminant T cell lymphoproliferative disease that arose in the setting of acute primary Epstein-Barr virus infection. Our patient, a 23-year-old man, presented to our facility with persisting fever, hepatosplenomegaly and severe pancytopenia. On bone marrow biopsy, an abundant lymphoid infiltrate was observed. Immunophenotypic and molecular studies revealed that the atypical lymphoid cells displayed a CD8^+^, Epstein-Barr-encoded-RNA-positive T cell phenotype with clonal rearrangement of the T cell receptor genes, the final diagnosis being systemic Epstein-Barr-virus-positive T cell lymphoproliferative disease. On reviewing the literature we found only 14 similar cases, all presenting with very aggressive clinical courses and requiring extensive phenotyping and molecular techniques for final diagnosis.

**Conclusion:**

Though extremely rare, this disease can occur in Europe, and a comprehensive diagnostic approach is thus recommended in all case of Epstein-Barr-virus-positive lymphoproliferative disorders. Unfortunately, at present no specific treatment is available; however, prompt administration of anti- Epstein-Barr virus treatment and rapid attempts to control the hemophagocytic syndrome are indicated.

## Introduction

Primary infection of Epstein-Barr virus (EBV) is commonly asymptomatic, but some children, adolescents and young adults develop infectious mononucleosis [1] (IM), a benign febrile disease characterized by hepatosplenomegaly, lymphadenopathy, and increase of activated CD8^+ ^T lymphocytes in peripheral blood [1,2]. However, exceptionally, younger patients can develop a very aggressive form, referred to in the past as 'fulminant infectious mononucleosis' or 'fatal haemophagocytic syndrome'. The disorder is characterized by rapid deterioration in previously healthy children, secondary to acute primary EBV infection; this syndrome is accompanied by high fever, skin rash, pulmonary infiltrate, jaundice, hepatosplenomegaly, cytopenia, haemophagocytic syndrome, and coagulopathy [3]. Unfortunately, patients commonly die within a few weeks of diagnosis.

In addition, EBV is implicated in the pathogenesis of different types of lymphoproliferative diseases (LPD), which are related to diverse immune alterations or peculiar clinical backgrounds [4]. Typically, EBV-associated lymphoproliferative disorders are derived from B cells, such as Hodgkin disease and Burkitt lymphoma, where memory B cells are the physiological reservoir of latent EBV [1]. Nonetheless, rare EBV-driven T cell tumors have been recognized.

In this regard, fulminant mononucleosis has recently been demonstrated to be a monoclonal CD8^+ ^LPD, and is currently classified as systemic EBV+ T cell LPD of childhood in the World Health Organization classification of tumors of hematopoietic and lymphoid tissues [5]. This entity is a rare clonal proliferation of EBV-infected T cells with an activated cytotoxic phenotype [5]; the disease occurs with increased frequency in immunocompetent children and young adults, appears to be more common in Asians and Native Americans, and is associated with rapid progression, high morbidity and mortality. It can develop after primary EBV infection or in association with chronic active EBV infection (CAEBV). Despite the name, the disease occurs not only in children but in adolescent and young adults as well, the median age being around 20 years [5].

At morphology, neoplastic T cells are usually small and lack significant cytological atypia [6]. However, cases with pleomorphic medium-sized to large-sized lymphoid cells, irregular nuclei and frequent mitoses have been described. The most typical phenotype is CD2^+^, CD3^+^, CD8^+^, CD56^-^, and TIA^+ ^[6-8]; conversely, cases arising in the setting of severe CAEBV are CD4^+^. Neoplastic cells have monoclonally rearranged T cell receptor (*TCR*) genes, and consistent Epstein-Barr encoded RNA (EBER) positivity at *in situ *hybridization (ISH). Differential diagnosis mainly concerns reactive conditions as well as aggressive natural killer (NK) cell leukemia.

Here, we report the clinicopathological features of fulminant T-LPD that arose in the setting of acute primary EBV infection in our patient, characterized by a monoclonal proliferation of EBV-infected T cells.

## Case presentation

A 23-year-old Caucasian man was hospitalized for persisting fever resistant to conventional therapies. On physical examination, our patient presented with marked hepatosplenomegaly and abnormal sounds at thoracic auscultation. Laboratory findings consisted of severe pancytopenia (hemoglobin 9.3 g/dL, platelets 93 × 10^9 ^cells/L, white blood cells 2.2 × 10^9 ^cells/L, neutrophils 410 × 10^9 ^cells/L, lymphocytes 1.570 × 10^9 ^cells/L), increased LDH, signs of disseminated intra-vascular coagulopathy (CID), and anti-EBV IgM positivity, while a chest X-ray showed diffuse pulmonary infiltrates. No prior immunological abnormalities were recorded.

**Figure 1:**
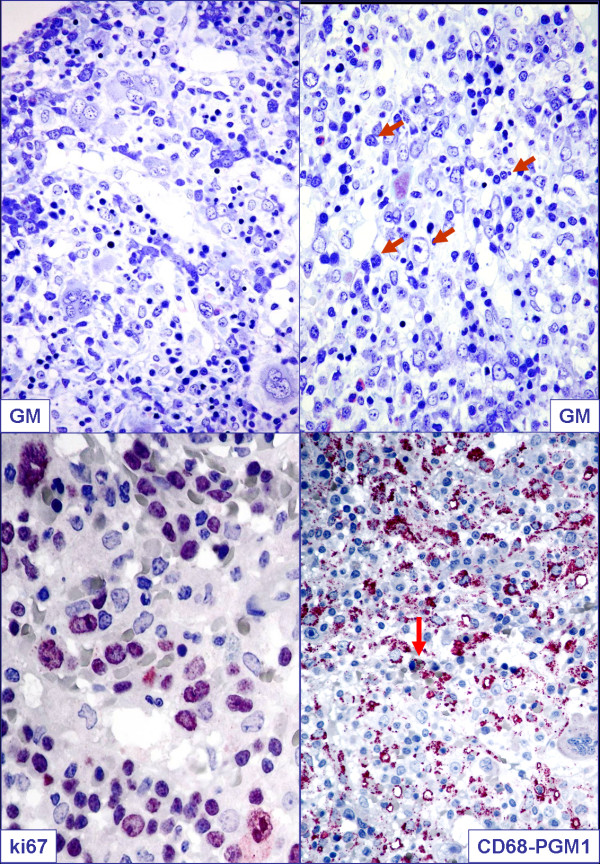
**Pathological findings on lymph node biopsy**. Giemsa, Ki67 and CD68 immunostains are shown. Arrows indicate atypical cells (GM) as well as eritrophagocytic syndrome (CD68).

**Figure 2:**
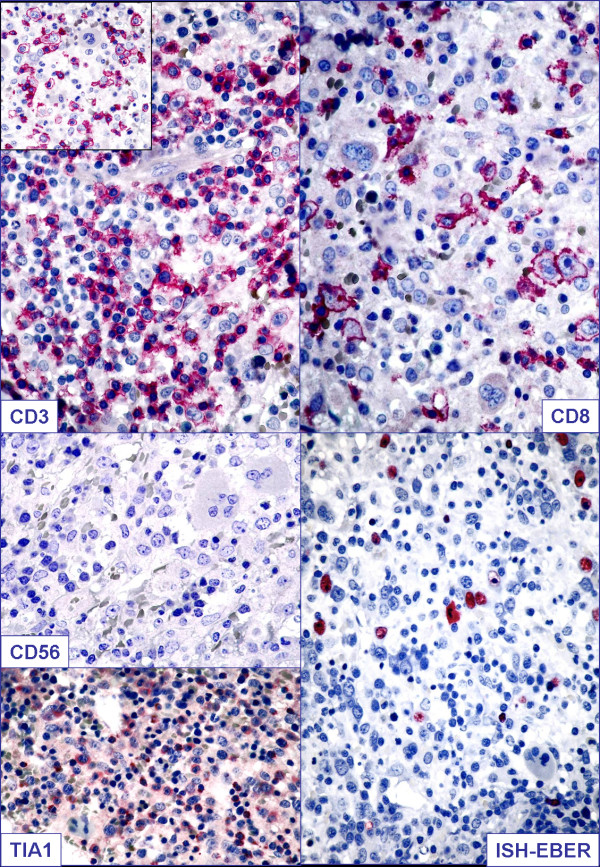
**Pathological findings on lymph node biopsy**. CD3, CD8, CD56, TIA1 immunostains and Epstein-Barr encoded RNA (EBER) *in situ *hybridization are shown.

For the suspicion of either massive bone marrow infiltration by leukemia/lymphoma or hemophagocytic syndrome a bone marrow biopsy was performed. Results from the biopsy showed the bone marrow was hypercellular, with numerous atypical lymphoid cells and occasional hemophagocytes (identified by positive staining for CD68/PGM1) (Figure 1). Lymphocytes were more often small and without significant atypia; a smaller percentage was represented by larger cells (Figures 1 and 2). Immunohistochemistry (IHC) investigation results showed atypical lymphocytes were CD79a^-^, CD3^+^, CD2^+^, CD8^+ ^and TIA1^+ ^(Figure 2). ISH for EBER demonstrated that the majority of lymphoid cells were positive (Figure 2). Finally, polymerase chain reaction (PCR) analysis revealed a monoclonal rearrangement of the *TCRγ *genes. IHC, ISH and molecular analyses were carried out as previously described [9,10].

Based on the above findings, a final diagnosis of systemic EBV+ T cell LPD of childhood was made. Our patient was initially treated with two sequential doses of VP16 with moderate improvement of his clinical and laboratory data. In particular, the fever transiently improved, hepatosplenomegaly was reduced, and coagulation parameters were partially corrected; however, severe peripheral blood cytopenia persisted. Soon after, the patient developed a fever recrudescence in association with pulmonary fungal infection.

A second bone marrow biopsy was performed, revealing (hypo)aplasia with a minimum percentage of CD79a^-^, CD3^+^, CD4^-^, CD8^+^, EBER^- ^small lymphocytes and absence of the previously observed CD8^+ ^large cells. VP16 was then replaced with cyclosporine, obtaining a white blood cell count increase and a further decline of splenomegaly, but with no improvement in thrombocytopenia. A third bone marrow biopsy showed an increased cellularity with reappearance of numerous CD8^+ ^lymphocytes and evident hemophagocytosis.

Our patient then developed rectal hemorrhages only treatable with surgery, which turned out to be sustained by microvascular thrombosis on histological examination. Finally, after a short period of relative good health, our patient had a relapse of rectal bleeding and died soon after with cerebral manifestations.

## Discussion

Systemic EBV+ T cell LPD of childhood is a rare disorder characterized by an aggressive disease course and dismal prognosis [5]. As death unfortunately often occurs within a few weeks, and at present there is no specific treatment, a prompt diagnosis is necessary. Our case report highlights the fact that, though rare, such a disease can occur also in Europe.

On reviewing the literature, we found only 14 cases reported in Western countries [6,11-14], specifically cases recorded in Europe and the USA (Table 1). Interestingly, the majority of cases have been reported in eastern Asia [5], specifically in Japan and Taiwan. The geographical distribution has been suggested to indicate possible genetically determined defects in T cell responses to EBV in certain populations.

**Table 1:** Cases of systemic Epstein-Barr virus positive (EBV^+^) T cell lymphoproliferative disease (LPD) of childhood described in Western countries

Reference	Age/sex	Race	Case description	Time to lymphoma	Histopathological features	TCR status	EBV status
Jones *et al*. [11]	two/M	Unspecified	Fever, generalized erythematous skin eruption, hepatosplenomegaly, pancytopenia, hypoplastic bone marrow, pulmonary infiltrates	six years	Pulmonary large cell lymphoma (phenotype: CD4^+^, HLA-DR^+^)	TCR-β rearranged	EBV^+^, clonal

	31/F	Unspecified	Fever, generalized lymphadenopathy, hepatosplenomegaly, pancytopenia, diarrhea, gastric pain	one year	Lymphoblastic lymphoma (phenotype: CD4^+^, HLA-DR^+^)	TCR-β γ rearranged	EBV^+^, clonal

	55/M	Unspecified	Gluten enteropathy for 19 years; fever, persistent diarrhea, nodular erythematous skin lesion	one year	Peripheral T cell lymphoma (phenotype: UCHL1^+^)	-	EBV^+^

Gaillard *et al*. [13]	seven/F	Unspecified	Infectious acute mononucleosis, persistent high-grade fever, weight loss, adenopathy, necrotizing skin lesions and VAHS	four months	Fulminant EBV^+ ^T cell LPD (phenotype: CD8^+^)	TCR-β γ rearranged	EBV^+^

Craig *et al*. [15]	20 months/F	Unspecified	Fever, generalized erythematous skin eruption, hepatosplenomegaly	-	T cell lymphoma NOS (phenotype: not interpretable)	TCR-β rearranged	EBV^+^, clonal

Quintanilla-Martinez *et al*. [6]	37/M	White	Fever, mental status of one week duration, hepatosplenomegaly, pancytopenia, jaundice	-	Fulminant EBV^+ ^T cell LPD (phenotype: CD4^+^, TIA1^+^)	TCR-γ rearranged	EBV^+^, clonal

	17/M	Native American	Symptoms of viral upper respiratory illness, hepatosplenomegaly, pancytopenia, jaundice	-	Fulminant EBV^+ ^T cell LPD (phenotype: CD8^+^, TIA1^+^)	TCR-γ rearranged	EBV^+^, clonal

	23/M	Asian	Fever, night sweats, weight loss, hepatosplenomegaly, pancytopenia, jaundice, generalized lymphadenopathy	-	Fulminant EBV^+ ^T cell LPD (phenotype: CD4^+^, CD8^+^, TIA1^+^)	TCR-γ rearranged	EBV^+^, clonal

	22/F	Native American	Fever weight loss, hepatosplenomegaly, jaundice	-	Fulminant EBV^+ ^T cell LPD (phenotype: CD4^+^, TIA1^+^)	Polyclonal	EBV^+^, clonal

	27 months/M	Native American	Fever, skin rash, hepatosplenomegaly, pancytopenia	-	Fulminant EBV^+ ^T cell LPD (phenotype: CD8^+^, TIA1^+^)	TCR-γ rearranged	EBV^+^, clonal

	15/F	White	IM at eight years old, followed by CAEBV. At 14 years old developed hepatosplenomegaly and hemophagocytic syndrome.	-	Fulminant EBV^+ ^T cell LPD (phenotype: CD4^+^, CD8^+^, TIA1^+^)	TCR-γ rearranged	EBV^+^, clonal

Wick *et al*. [14]	12/M	Unspecified	Hemophagocytic syndrome, FIM	-	Fulminant EBV^+ ^T cell LPD (phenotype: not reported)	TCR-β γ rearranged	EBV^+^, clonal

	three/F	Unspecified	Hemophagocytic syndrome, FIM	-	Fulminant EBV^+ ^T cell LPD (phenotype: not reported)	TCR-β rearranged	EBV^+^, clonal

	nine/M	Unspecified	Hemophagocytic syndrome, FIM	-	Fulminant EBV^+ ^T cell LPD (phenotype: not reported)	TCR-β rearranged	EBV^+^, biclonal

CAEBV = chronic active EBV infevtion; FIM = fatal infectious mononucleosis; HLA = human leukocyte antigen; IM = infectious mononucleosis; NOS = not otherwise specified; TCR = T cell receptor; VAHS = virus-associated hemophagocytic syndrome.

Our review of Western cases showed that four of those 14 patients developed a T cell LPD after CAEBV infection [6,11], and 10 presented a fulminant EBV T cell LPD following acute EBV infection. In the former group, ethnic origin was specified only in one case (white); in the latter group, one patient was of Caucasian descent, four were Asian or Native American, while in five cases the ethnic group was not specified. Mean age at onset was 17 years and the male/female ratio was 2.3:1. Common symptoms were fever, hepatosplenomegaly and hematophagocytic syndrome; the clinical courses were fulminant in patients with T cell LPD after acute IM. In particular, in the acute IM group three cases had a CD8^+ ^phenotype, two a CD4^+ ^phenotype and one showed double positivity for CD8 and CD4; in one case phenotype was not interpretable and in three cases it was not reported. *TCR*α presented with clonal rearrangements in nine out of 10 patients and EBV genome was clonal in all but one case. In contrast, among patients with T cell LPD after CAEBV infection two were CD4^+^, one was CD45RO^+ ^and one presented with an admixture of CD8^+ ^and CD4^+ ^lymphocytes; three cases presented with monoclonal patterns with regard to rearrangement of both *TCR*α genes and EBV genome. In the remaining case, only the EBV positivity was assessed.

In all described cases, an accurate diagnostic investigation including clinical, morphological, immunohistochemical, and molecular analyses was necessary in order to formulate a correct diagnosis. In particular, the differential diagnosis with aggressive NK cell leukemia was based on surface sCD3 and CD8 positivity, CD56 negativity, and evidence of *TCR*α rearrangement in systemic EBV^+ ^T cell LPDs, and also sCD3/CD8 negativity, CD56 positivity and germline *TCR*α patterns in aggressive NK cell leukemia cases.

## Conclusion

In conclusion, our case report underlines the importance of a comprehensive diagnostic approach in the management of atypical EBV^+ ^LPDs. In fact, though, at present, specific therapies are not available, the correct description of rare disorders is essential for improving current knowledge and possibly future therapeutic approaches.

## Consent

Written informed consent was obtained from the patient for publication of this case report and any accompanying images. A copy of the written consent is available for review by the Editor-in-Chief of this journal.

## Competing interests

The authors declare that they have no competing interests.

## Authors' contributions

VT performed research, analyzed data and wrote the manuscript; CA performed research and analyzed data; ES and FB analyzed data; SC and GM were responsible for patient care and provided clinical information; AG, CM, SR, and MTS analyzed data; SAP and PPP performed research, analyzed data and wrote the manuscript. All authors read and approved the final manuscript. VT and CA contributed equally to this work; SAP and PPP contributed equally to this work.
